# Case of Rapidly Fatal Cholera-Morbus, from the Presence of Crude Food in the Bowel

**Published:** 1855-07

**Authors:** J. Forsyth Meigs


### *Case of rapidly fatal Cholera-Morbus, from the presence of crude food in the bowel.* By J. Forsyth Meigs, M.D.

Miss M , æt. 55, the subject of the following case, had had rather feeble health for some years, being subject to frequent attacks of dyspepsia and diarrhœa. Her general appearance was frail, but there was no evidence of positive organic disease of the great organs. She had had for a number of years double femoral hernia.

I was sent for at 9 P. M., of May 18th, 1855. She had been as well as usual in the morning, and was busily occupied about the house until between 2 and 3 P. M., when she was seized with considerable pain in the abdomen, and some diarrhœa. Nevertheless, she continued actively engaged in house-cleaning until 5 P. M., when she went to bed complaining very much of severe and griping abdominal pain, with frequent desire to go to stool. The dejections were very small. During this time she had, also, a great deal of nausea and retching, but rejected only small quantities of fluid from the stomach. At 9½ P. M., when I saw her, her countenance looked badly. The first impression it gave me (and correctly too, as the event proved,) was that she was seriously ill. The face was pale, anxious, somewhat pinched, and, in fact, choleratic. The pulse, however, was of good volume, not weak, regular, and only 92 in the minute. The skin was natural, neither too warm nor too hot. The tongue was moist and clean. On examining the abdomen, I found the lower half tumid, rounded, quite tympanitic, and somewhat, but not very tender to the touch. The muscular force was good, as she turned freely in bed at my request, and had been rising often to the sick-chair. There was no fault in the intelligence or senses, and she expressed herself as not feeling seriously ill. There had been some ten or fifteen discharges by stool during the afternoon, some of which I saw, and found them to consist of small quantities of mucus and blood. I could not learn that there had been any indiscretion in diet, or any other cause for the attack.

Supposing the case to be one of sudden dysentery, I ordered a large teaspoonful of castor oil with ten drops of laudanum, and some pills of opium and gallic acid, to be taken afterwards. A laudanum injection was to be given if necessary.

I did not see the patient again, as she died very suddenly in the night, but learned, upon inquiry, that the discharges ceased almost entirely soon after my visit, though there was still frequent desire to go to stool. The pain in the abdomen was violent, at times intense. About 1 o'clock in the night her hands were observed to be very blue, and her mind wandered a little. At 1½ o'clock she suddenly expired, just after having been raised up in bed momentarily, at her own request, in the hope of alleviating her suffering. As she was dying, there was rejected from the stomach a very large amount of thin liquid of a dark color.

At the post-mortem examination, on the following day, the appearances were as follows: Abdomen very much distended, and highly tympanitic. On opening that cavity the distention was found to occupy chiefly the small intestines, whilst the large was rather diminished in size. A small amount of reddish serous effusion was observed in the posterior part of the peritoneal cavity. No traces of peritoneal inflammation. On each side was the sac of an old femoral hernia, but entirely empty, and without a sign of inflammatory action. Stomach large, much inflated with gas, and containing a very large quantity of pinkish serous fluid; its coats, especially about the pylorus, softened. Small intestine greatly distended down to within about eight inches of the cœcum, and filled with gas, and particularly with enormous quantities of a thin, watery serum, colored of a marked pinkish tint. The amount of this fluid was immense, it being found at all parts of the small intestines, and rushing out in a full stream wherever these were opened. The mucous membrane was healthy, being of the proper color and consistence. Shining through the peritoneal coat, however, as the intestine was viewed from without, there was, here and there, a somewhat marked arborescence. Eight inches above the ileo-cœcal valve was found a foreign body, filling up rather completely, but not entirely obstructing, the calibre of the intestine. In the immediate neighborhood of this body the mucous membrane was slightly injected, somewhat softened, and the isolated glands were a little enlarged. The foreign body consisted of a portion of apple scarcely at all softened by the alimentary fluids. A thin layer on the outside was alone softened, while the interior was scarcely moist even. The piece of apple consisted of about one-third of a medium sized one. It measured about two inches in breadth, and the same in length.

Below the point where the apple was found the intestinal canal was healthy. The only deviation from the natural appearances was an unusual contraction at some parts, in the diameter of the tube. None of the purplish serum above spoken of was found below the point where the apple was lodged.

*Remarks.*—The above case was clearly one of cholera-morbus caused by the presence of crude food in the intestinal tube. The piece of apple which caused the death of the patient was too large to have been swallowed in the form in which it was found. It must have been swallowed as dried apple, in which state, having lost all its fluids, it would not possess more than a fourth or sixth of the bulk it did in the recent state. This case is, therefore, still another instance of the frail tenure by which life is held, and another proof that the health, and even the life of the human being is, to some extent, at least, within his own control. Had this woman been taught more caution about her eating, had she been taught even a little physiology in her younger days, she had never swallowed a mass of hard, coriaceous, dried fruit, which, at the best, would task severely the solvent power of her stomach, and at the worst, might pass undissolved into the intestine, and there so irritate and disturb the order of vitality as to cause death in the short space of less than a single day.

